# Biliary Secretion of Quasi-Enveloped Human Hepatitis A Virus

**DOI:** 10.1128/mBio.01998-16

**Published:** 2016-12-06

**Authors:** Asuka Hirai-Yuki, Lucinda Hensley, Jason K. Whitmire, Stanley M. Lemon

**Affiliations:** a Lineberger Comprehensive Cancer Center, the University of North Carolina at Chapel Hill, Chapel Hill, North Carolina, USA; b Department of Microbiology & Immunology, the University of North Carolina at Chapel Hill, Chapel Hill, North Carolina, USA; c Department of Genetics, the University of North Carolina at Chapel Hill, Chapel Hill, North Carolina, USA; d Department of Medicine, the University of North Carolina at Chapel Hill, Chapel Hill, North Carolina, USA

## Abstract

Hepatitis A virus (HAV) is an unusual picornavirus that is released from cells cloaked in host-derived membranes. These quasi-enveloped virions (eHAV) are the only particle type circulating in blood during infection, whereas only nonenveloped virions are shed in feces. The reason for this is uncertain. Hepatocytes, the only cell type known to support HAV replication *in vivo*, are highly polarized epithelial cells with basolateral membranes facing onto hepatic (blood) sinusoids and apical membranes abutting biliary canaliculi from which bile is secreted to the gut. To assess whether eHAV and nonenveloped virus egress from cells via vectorially distinct pathways, we studied infected polarized cultures of Caco-2 and HepG2-N6 cells. Most (>99%) progeny virions were released apically from Caco-2 cells, whereas basolateral (64%) versus apical (36%) release was more balanced with HepG2-N6 cells. Both apically and basolaterally released virions were predominantly enveloped, with no suggestion of differential vectorial release of eHAV versus naked virions. Basolateral to apical transcytosis of either particle type was minimal (<0.02%/h) in HepG2-N6 cells, arguing against this as a mechanism for differences in membrane envelopment of serum versus fecal virus. High concentrations of human bile acids converted eHAV to nonenveloped virions, whereas virus present in bile from HAV-infected *Ifnar1*^*−/−*^ *Ifngr1*^*−/−*^ and *Mavs*^*−/−*^ mice banded over a range of densities extending from that of eHAV to that of nonenveloped virions. We conclude that nonenveloped virions shed in feces are derived from eHAV released across the canalicular membrane and stripped of membranes by the detergent action of bile acids within the proximal biliary canaliculus.

## IMPORTANCE

HAV is a hepatotropic, fecally/orally transmitted picornavirus that can cause severe hepatitis in humans. Recent work reveals that it has an unusual life cycle. Virus is found in cell culture supernatant fluids in two mature, infectious forms: one wrapped in membranes (quasi-enveloped) and another that is nonenveloped. Membrane-wrapped virions circulate in blood during acute infection and are resistant to neutralizing antibodies, likely facilitating HAV dissemination within the liver. On the other hand, virus shed in feces is nonenveloped and highly stable, facilitating epidemic spread and transmission to naive hosts. Factors controlling the biogenesis of these two distinct forms of the virus in infected humans are not understood. Here we characterize vectorial release of quasi-enveloped virions from polarized epithelial cell cultures and provide evidence that bile acids strip membranes from eHAV following its secretion into the biliary tract. These results enhance our understanding of the life cycle of this unusual picornavirus.

## INTRODUCTION

Hepatitis A virus (HAV) is an unusual member of the *Picornaviridae* family. Its capsid differs structurally from that of other mammalian picornaviruses, with a VP2 domain swap found only in insect-resident members of the *Picornavirales* (1). The capsid also has both unusually high physical stability and a distinct assembly mechanism (2, 3). Strongly hepatotropic, HAV is spread by fecal-oral transmission and causes acute inflammatory liver disease in humans (4). While the capsid of virus shed in feces of infected individuals is naked and nonenveloped, virions circulating in the blood during acute infection are completely enveloped in host-derived membranes that provide protection from neutralizing antibodies directed against the capsid (5). These quasi-enveloped virions (eHAV) are infectious and are similar to exosomes in both size and buoyant density. They share several attributes with classical enveloped viruses, although there is no evidence for the presence of virally encoded peplomers on their surface (5, 6).

The mechanisms underlying the dramatic differences in the physical characteristics of virus in the blood (where it is entirely quasi-enveloped) versus the feces (where it is naked and nonenveloped) are not clear. Some data suggest that HAV undergoes limited replication within the gastrointestinal tract (7). However, the principal site of replication is believed to be the hepatocyte, with the virus that is shed in feces being secreted to the gastrointestinal tract from the liver through the biliary system. Consistent with this, large numbers of nonenveloped virions have been visualized in the bile of experimentally infected chimpanzees (8). A similar situation exists in mice with defects in type I interferon signaling that are permissive for infection by human HAV (9). Replication is restricted to hepatocytes in these mice, which have substantial fecal shedding of nonenveloped HAV virions despite little if any detectable viral RNA in tissues of the small and large intestine (9).

**FIG 1:**
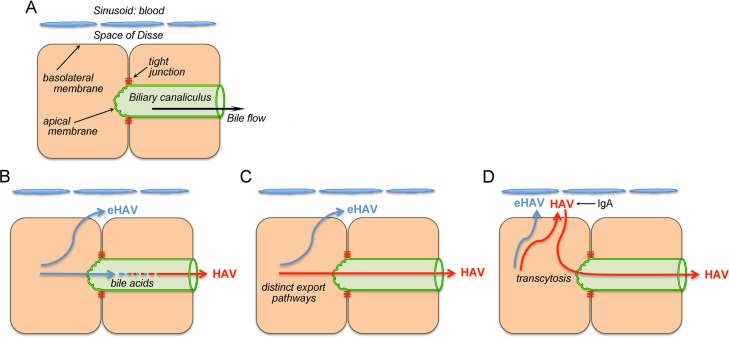
Microanatomy of the hepatocyte and three possible explanations for the presence of different forms of HAV in blood (quasi-enveloped) and feces (naked, nonenveloped). (A) Simplified diagram showing two hepatocytes in relation to the space of Disse and hepatic sinusoid that is bathed in blood and to the biliary canaliculus that encircles hepatocytes in a belt-like fashion and into which virus and bile are secreted across the hepatocyte apical membrane. Bile flows from the canaliculus to larger bile ductules and ultimately to the lumen of the gastrointestinal tract. (B to D) Competing hypotheses that could explain differences in membrane association and quasi-envelopment of virus in different compartments. See introductory text for details.

Hepatocytes are highly polarized cells of epithelial origin with distinct basolateral and apical membranes and very specialized protein export pathways (10, 11). Their basolateral membrane faces onto the space of Disse, which communicates with blood flowing through the hepatic sinusoids, whereas the smaller apical membrane abuts the lumen of the biliary canaliculus (Fig. 1A). Bile acids are secreted by hepatocytes across the apical membrane into the canaliculus and then flow toward larger bile ductules to eventually reach the gut (11). Tight junctions forming between neighboring hepatocytes separate apical and basolateral membranes and seal the canalicular lumen, forming a barrier between blood and bile that prevents diffusion of bile acids and other large solutes.

Several hypotheses can explain why quasi-enveloped eHAV virions are found in the circulation whereas nonenveloped virus is shed from the gastrointestinal tract. The simplest explanation would be that eHAV membranes are eliminated by the detergent action of bile acids during passage through the biliary tract (Fig. 1B). Previous studies have shown that the eHAV membrane is stable when virus is incubated for 2 h at 37°C in 98% freshly collected porcine bile (reference 5 and unpublished observations). However, bile acid concentrations are greater and their detergent action is much stronger in the proximal biliary canaliculus where bile originates, making this hypothesis a continuing possibility (11).

An alternate hypothesis is that the two forms of the virus egress from infected hepatocytes via distinct export pathways, with eHAV released into the blood and nonenveloped virions into the bile (Fig. 1C). Previous studies have shown HAV to be secreted in a vectorial fashion from polarized epithelial cell cultures (12, 13), but those studies did not differentiate between eHAV and nonenveloped virions, leaving open the possibility of differential release of eHAV versus HAV. Yet a third hypothesis, suggested recently by Counihan and Anderson (14), proposes that both quasi-enveloped and nonenveloped virions are released across the basolateral membrane into the space of Disse but that the naked, nonenveloped virions subsequently reenter hepatocytes and undergo transcytosis to the apical membrane, where they are released into the biliary tract (Fig. 1D). Late in the course of infection, transcytosis may be enhanced by IgA antibodies binding the HAV capsid (14). While seemingly complicated, this proposed apical export pathway mimics some aspects of the default pathway utilized by hepatocytes for transport of apical proteins (10, 11).

Here, we describe a series of experiments in cell culture and HAV-infected mice that showed that HAV is released across both the basolateral and apical membranes of polarized epithelial cells predominantly (if not exclusively) as enveloped eHAV virions. We show that high concentrations of human bile acids effectively convert quasi-enveloped eHAV virions to naked, nonenveloped particles and demonstrate the presence of virions undergoing this transition in bile collected from infected mice.

## RESULTS

### Vectorial release of virus from polarized Caco-2 cells.

**FIG 2:**
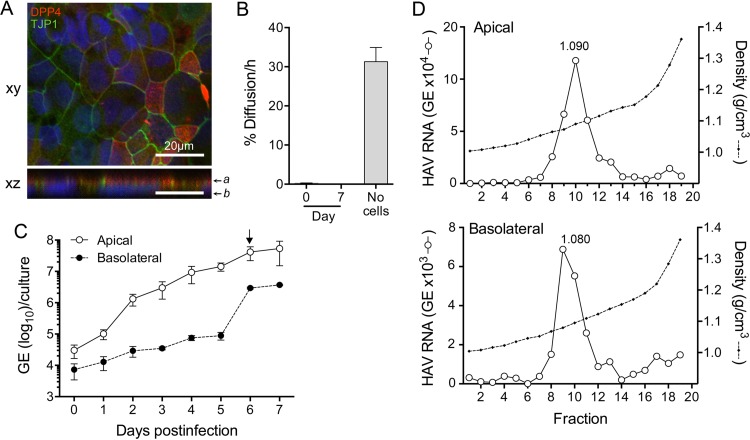
HAV infection of polarized Caco-2 cell monolayers. (A) Polarity was assessed by determining the intracellular localization of DPP4 (CD26) and TJP1 (ZO-1). Cells were seeded into chamber slides and cultured for 10 days before being fixed and stained with antibodies specific for DPP4 (red) and TJP1 (green). Nuclei were counterstained with DAPI (blue). Images of *xy* and *xz* sections were collected by confocal microscopy. *z* sections were compiled by taking 0.41-μm steps through each *x*-*y* section; “*a*” and “*b*” indicate approximate positions of apical and basolateral membranes, respectively. The scale bars represent 20 μm. (B) The integrity of monolayer cell cultures grown on Transwell membranes was assessed by measuring paracelluar diffusion of FITC-dextran prior to (day 0) and 7 days after virus infection. Results shown represent the mean percentage ± range of FITC-dextran delivered to the apical chamber that was detected in the basolateral chamber 60 min later (see Materials and Methods). (C) Vectorial release of quasi-enveloped HAV from polarized Caco-2 cells. After 14 days of culture on Transwell inserts, cells were infected by inoculating the apical surface with HAV. Total apical and basolateral fluids were collected at 24-h intervals, and HAV RNA was quantified by RT-qPCR. (D) The buoyant density of virus particles present in the apical (top) and basolateral (bottom) compartments of Transwell cultures on day 6 of the infection was determined by centrifugation in isopycnic iodixanol gradients followed by HAV-specific RT-qPCR.

In previous studies, we found that most (~80%) virions present in supernatant fluids of infected Huh-7.5 human hepatoma cells were quasi-enveloped, whereas the remainder were naked, nonenveloped particles (5). To determine whether differences exist in the modes of vectorial release of these two particle types, we studied Caco-2 cells, a human colonic adenocarcinoma-derived cell line that is known to establish a high degree of polarity in culture and is widely used as a model system for intestinal epithelial permeability (15–17). Caco-2 cells exhibit a simple polarity consisting of single apical and basolateral surfaces that oppose each other, and previous work in our laboratory has shown that they release HAV almost exclusively from the apical membrane (12). We confirmed the polarity of cells grown on glass slides by assessing localization of the apical membrane protein dipeptidyl-peptidase 4 (DPP4, or CD26) and tight junction protein 1 (TJP1, or ZO-1) by confocal immunofluorescent microscopy (Fig. 2A). TJP1 was present at intercellular contacts, ringing the perimeter of cells in *x*-*y* sections and thus confirming the existence of intact tight junctions (Fig. 2A, top). In contrast, DPP4 localized exclusively to the apical cell surface in *x*-*z* sections (Fig. 2A, bottom), confirming that the Caco-2 cells form polarized monolayers.

To assess the integrity of Caco-2 monolayers grown on a permeable membrane (Transwell-COL insert), we measured the diffusion of fluorescein isothiocyanate-conjugated dextran (FITC-dextran; 500 kDa) placed in the upper (apical) chamber of the insert into the lower (basolateral) chamber over time. In the absence of cells, the diffusion rate was 31.3%/h (Fig. 2B). This was reduced to 0.27%/h by the Caco-2 monolayer, which is below the (^3^H)inulin (monomer mass of 5 kDa) diffusion rate of <1.0%/h considered indicative of the formation of intact tight junctions (16, 18). The molecular weight of a tracer is likely to influence its diffusion rate, with high-molecular-weight tracers diffusing slowly compared to those with low molecular weight. Since the diameters of nonenveloped (27 nm) and quasi-enveloped (~50 to 110 nm) virions (5) are similar to or larger than that of FITC-dextran (29 nm) (19), these results indicated that paracellular diffusion was unlikely to bias measurements of vectorial virus release from these cells (5).

To examine vectorial release, Caco-2 cell monolayers were apically infected with HAV, as HAV entry occurs efficiently at the apical surface of these cells (12). The virus that we used, HM175/p16, is adapted to growth in cell culture and relatively attenuated in its capacity to cause disease in primates and causes no detectable cytopathic effect in cell culture (20, 21). The FITC-dextran diffusion rate was not increased by infection over a period of 7 days (Fig. 2B), indicating that barrier function was not disturbed. Apical and basolateral fluids were collected daily over this period, and the quantity of virus released into each compartment was measured by real-time quantitative PCR (RT-qPCR). Virus was released almost (>99%) exclusively from the apical membrane during the period of exponential growth (day 4 to day 5), reaching 5.4 × 10^7^ genome equivalents (GE)/culture by day 7 (Fig. 2C). The level of virus release across basolateral membranes rose slowly until day 5 but then exponentially increased to 2.9 × 10^6^ GE/culture at day 6, reaching a maximum of 4.2% to 12.7% of all virus released on days 6 to 7. These results were replicated in independent experiments and are consistent with a previous report from our laboratory showing that HAV release is largely restricted to the apical domain of Caco-2 cells (12). To determine the proportion of quasi-enveloped eHAV virions released from apical versus basolateral domains, supernatant fluids from day 6 were concentrated and eHAV and nonenveloped HAV populations separated on an isopycnic gradient (see Materials and Methods). The density of the quasi-enveloped virions in isopycnic iodixanol gradients is approximately 1.08 g/cm^3^, whereas nonenveloped particles band at densities greater than 1.22 g/cm^3^ (5). Virus released across both the apical and basolateral membranes of Caco-2 cells banded predominantly at the density of quasi-enveloped virions (fractions 8 to 12; 1.065 to 1.115 g/cm^3^) (Fig. 2D). Only a small proportion of virus banded at a density consistent with nonenveloped particles (fractions 17 to 19; 1.213 to 1.341 g/cm^3^), indicating a much lower abundance of nonenveloped virions in these samples. It is not clear whether these nonenveloped virions were released as such from the cells or were derived from eHAV particles that had sustained loss of the enveloping membrane during concentration by ultrafiltration followed by high-speed centrifugation through an iodixanol gradient.

### Vectorial release of virus from polarized HepG2-N6 cells.

**FIG 3:**
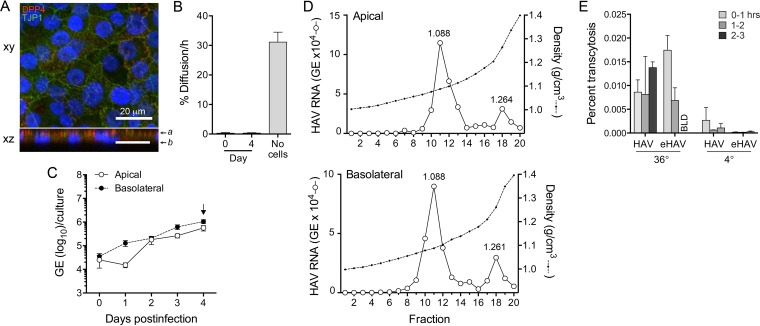
HAV infection of polarized HepG2-N6 cell monolayers. (A) Polarity was assessed by determining the intracellular localization of DPP4 and TJP1. Cells were cultured for 17 days before being fixed and stained with specific antibodies. See the legend to Fig. 1A for additional details. (B) Paracellular diffusion of FITC-dextran in monolayer cultures of HepG2-N6 cells. See the legend to Fig. 2B for details. (C) Vectorial release of virus from HepG2-N6 cells. Cells were infected at the basolateral surface after 18 days culture on Transwell membranes. Total apical and basolateral fluids were subsequently collected at 24-h intervals. HAV RNA was quantified by RT-qPCR. (D) Buoyant densities of virions present in the apical (top) and basolateral (bottom) compartments of Transwell cultures 4 days after infection. (E) Basolateral to apical compartment transcytosis of nonenveloped (“naked”) and enveloped eHAV virions by HepG2-N6 cells. Gradient-purified HAV and eHAV (1 × 10^8^ GE) were added to the basolateral compartment of polarized cultures of HepG2-N6 cells maintained at either 36°C or 4°C. Virus released into the apical chamber at hourly intervals was quantified by HAV-specific RT-qPCR. Results are shown as the mean percentage ± range of virus added to the basolateral chamber that underwent transcytosis in duplicate cultures during the period indicated. BLD, below the level of detection.

The N6 clone of the HepG2 human hepatocellular carcinoma cell line (HepG2-N6 cells) has been reported to exhibit simple polarity, with single apical and basolateral surfaces that oppose each other, and to support vectorial release of HAV (13, 22). However, unlike Caco-2 cells, which release virus apically, these cells were reported to release HAV predominantly across the basolateral membrane (13). To confirm polarity, HepG2-N6 cells were cultured on glass slides for 17 days and then stained for DPP4 and TJP1 (Fig. 3A). As with the polarized Caco-2 cells, DPP4 was localized to the apical cell surface, while TJP1 displayed a ring-like pattern in *xy* sections. By light microscopy, there was no suggestion of the formation of the biliary canaliculus-like cysts or ductules which the apical surfaces of adjacent cells form in primary hepatocytes and those of differentiated hepatic cell lines form in culture (10), although this has been reported for HepG2 cells previously (23). These results confirm that HepG2-N6 cells form simple polarized monolayers with a columnar orientation not dissimilar from that of Caco-2 cells. The rate of FITC-dextran diffusion was only 0.30%/h across HepG2-N6 monolayers grown on Transwell inserts and was not increased by HAV infection over a 4-day period (Fig. 3B).

HepG2-N6 cell monolayers were infected with HAV via the basolateral rather than the apical membrane, since HAV reportedly enters HepG2-N6 cells more efficiently via this domain (13). Apical and basolateral fluids were collected daily for 4 days, and the quantity and type of virus present in each compartment were assessed as described for the Caco-2 cells. Replication was much less efficient than in Caco-2 cells, with lower maximal yields (compare Fig. 3C with Fig. 2C). Increasing amounts of virus were released into the basolateral fluid continuously from day 1, whereas an increase in apically released virus did not begin until day 2 (Fig. 3C). This delay in apical virus release was reproduced in an independent, repeat experiment. At 1 day after infection, 87.5% of all released virus was directed into basolateral culture fluids, consistent with the report by Snooks et al. (13) that virus is predominantly (over 90%) secreted from the basolateral membrane of HepG2-N6 cells. Virus release was more balanced on day 2 and thereafter, although there was always greater release into the basolateral rather than the apical compartment (mean, 64% versus 36%; *P* < 0.001 by two-way analysis of variance [ANOVA]).

We collected day 4 culture supernatants to assess the buoyant density of the extracellular virus. Virus present in both apical and basolateral compartments was again predominantly enveloped (80.5% and 77.5%, respectively), banding at a density of 1.064 to 1.125 g/cm^3^ (fractions 9 to 13) (Fig. 3D). However, in contrast to the Caco-2 cells, a substantial minor population of nonenveloped virions was present in both compartments (fraction 18; 1.261 g/cm^3^). The relative proportions of quasi-enveloped versus nonenveloped virions were equivalent in the apical and basolateral compartments of the HepG2-N6 cultures (Fig. 3D). Collectively, these results argue against the hypothesis that eHAV and nonenveloped HAV particles are released from cells via vectorially distinct export pathways (Fig. 1C).

The reproducible 1-day delay that we observed in apical release of HAV from polarized cultures of HepG2-N6 cells (Fig. 3C) suggested the possibility that apical viral egress might result from transcytosis of virions released into the basolateral compartment (Fig. 1D). To assess this possibility, we added either gradient-purified eHAV or nonenveloped virions to the basolateral compartment of polarized HepG2-N6 monolayers grown on Transwell inserts and then measured the transport of virus into the apical compartment over the ensuing 3 h (see Materials and Methods). Somewhat greater amounts of virus were recovered from the apical compartment when cells were incubated at 36°C versus 4°C (*P* = 0.065 by two-sided Mann-Whitney test) (Fig. 3E), consistent with a low level of energy-dependent transcytosis. Overall, however, there was very limited basolateral to apical transport of HAV under these conditions and there were no significant differences between eHAV and nonenveloped virions (Fig. 3E). The maximum basolateral to apical transport of virus was observed with eHAV at between 0 and 1 h, but the virus released into the apical compartment represented only 0.015% of the virus added to the basolateral compartment. We conclude from these results that basolateral to apical transcytosis of HAV, while possibly present, does not contribute significantly to the release of virus from apical membranes of polarized HepG2-N6 cells.

### Bile acid treatment of eHAV.

**FIG 4:**
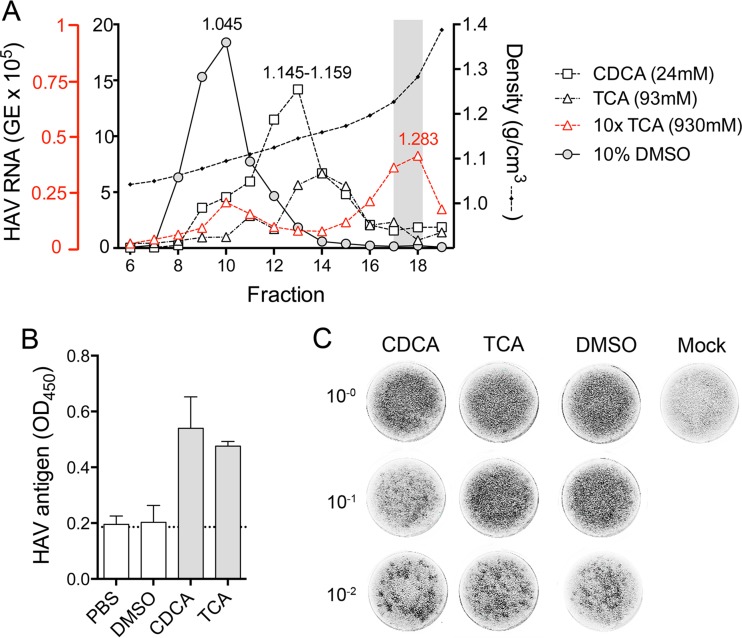
Stability of quasi-enveloped eHAV in human bile acids. (A) Quasi-enveloped eHAV virions were incubated with chenodeoxycholic acid (CDCA; 24 mM), taurocholic acid (TCA; 93 mM or 930 mM), or DMSO (10%) at 37°C for 2 h. The virus was then centrifuged to equilibrium in isopycnic iodixanol gradients. The buoyant density of virions was determined by measuring the quantity of HAV RNA in fractions 6 to 19 by RT-qPCR. The distribution of virus in the DMSO-treated sample matched that of virus incubated in PBS alone (not shown). The shaded zone indicates the expected density of nonenveloped HAV virions. (B) HAV capsid antigen ELISA of gradient-purified eHAV following treatment with bile acids. Virus samples (approximately 2 × 10^7^ GE) were subjected to diafiltration to remove bile acids and then tested for the presence of detectable (exposed) capsid antigen by ELISA (see Materials and Methods). (C) Infectivity of eHAV following treatment with bile acids. Samples were subjected to diafiltration to remove bile acids and then diluted and inoculated onto FRhK-4 cells for IR-FIFA. The content of the 10^−2^ dilution of the CDCA-treated sample was 3.8 × 10^4^ GE/ml, that of the 10^−2^ dilution of the TCA-treated sample was 3.1 × 10^4^ GE/ml, that of the 10^−2^ dilution of the DMSO-treated sample was 2.3 × 10^4^ GE/ml, and that of the 10^−2^ dilution of the PBS-treated sample was 3.6 × 10^4^ GE/ml.

In previous studies, the buoyant density of eHAV was not altered by incubation in 90% to 98% porcine bile for 2 h at 37°C (reference 5 and unpublished data). However, these conditions poorly recapitulate those on the luminal side of the apical canalicular membrane of human hepatocytes across which both bile acids and virus are transported (10, 11) (Fig. 1A). Although never measured because of anatomical constraints, the concentration and hence detergent action of bile acids is greatest on the luminal surface of the canalicular membrane. Surrounding hepatocytes in a belt-like manner, the apical membrane is rich in sphingomyelin, relatively resistant to the detergent action of bile acids, and highly active metabolically. Bile acids secreted across the membrane by apical transporter proteins are progressively diluted, and their detergent action is buffered by water and phospholipid secretion, leading to micelle formation. Moreover, the concentrations and types of bile acids secreted differ considerably among mammalian species and are different in humans and pigs (24). We therefore assessed the stability of the quasi-envelope of eHAV in the presence of physiologically relevant concentrations of chenodeoxycholic acid (CDCA) and taurocholic acid (TCA), the most abundant bile acids present in human bile (11, 24). Gradient-purified eHAV was incubated in 24 mM CDCA, 93 mM TCA, or solvent control (10% dimethyl sulfoxide [DMSO]) for 2 h at 37°C, and its buoyant density was then determined by isopycnic ultracentrifugation in iodixanol gradients. Compared to the solvent control results, CDCA treatment induced a shift in the peak location of eHAV in the gradients from fractions 9 and 10 (1.072 to 1.087 g/cm^3^) to fractions 12 and 13 (1.148 to 1.154 g/cm^3^) (Fig. 4A). TCA treatment induced a more pronounced shift to fractions 13 to 15 (1.146 to 1.175 g/cm^3^). Thus, concentrations of bile acids typical of human bile significantly increase the buoyant density of eHAV, consistent with partial disruption of the membranes enveloping the virus. A higher concentration of TCA (930 mM) fully converted quasi-enveloped eHAV to the density of nonenveloped virions (fractions 17 and 18; 1.227 to 1.283 g/cm^3^). Viral capsid antigen was detected by enzyme-linked immunosorbent assay (ELISA) in eHAV preparations after but not before treatment with CDCA (24 mM) or TCA (93 mM), indicating that the eHAV membrane was destroyed by the bile acids (Fig. 4B). We also assessed the infectivity of the virus after such treatment, removing the bile acids by diafiltration prior to titration by infrared fluorescent immunofocus assay (IR-FIFA). Despite their effect on buoyant density, these concentrations of CDCA and TCA did not reduce the infectivity of eHAV (Fig. 4C).

### Buoyant density of virions in bile from HAV-infected mice.

The recent discovery that wild-type human HAV replicates efficiently in *Ifnar1*^*−/−*^ and *Mavs*^*−/−*^ mice has made it possible to study the trafficking of virus in a readily accessible small-animal model (9). Infected *Ifnar1*^*−/−*^ mice, lacking expression of the type 1 interferon receptor, develop acute hepatic inflammation associated with hepatocellular apoptosis and elevated serum alanine aminotransferase (ALT) activity, whereas infected *Mavs*^*−/−*^ mice replicate the virus to higher levels but show no evidence of disease (9). Viral replication appears to be limited to hepatocytes in both knockouts (KOs). Abundant virus is shed in the feces of both infected *Ifnar1*^*−/−*^ and infected *Mavs*^*−/−*^ mice. This is likely to occur via biliary secretion, as in humans and chimpanzees, because very little if any viral RNA can be detected in ileal or colonic tissues. As in primates, the virus shed in feces by infected mice is entirely nonenveloped (1.230 g/cm^3^) (9).

**FIG 5:**
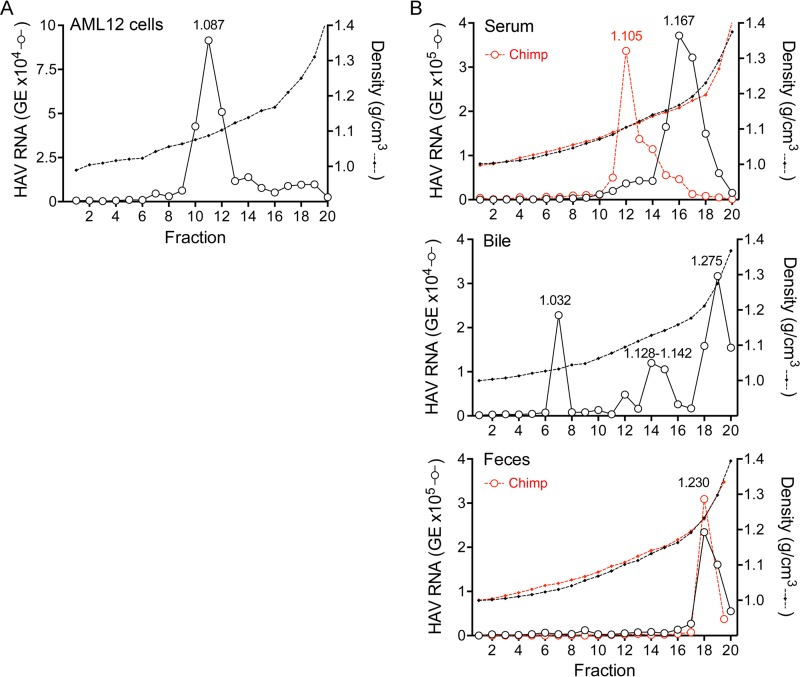
Buoyant densities of virions in serum and bile from infected *Mavs*^*−/−*^ mice. (A) Supernatant fluids from infected AML12 cells were concentrated by filtration and subjected to iodixanol density gradient centrifugation. The buoyant density of virions was determined by measuring the quantity of HAV RNA in fractions by RT-qPCR. (B) Buoyant densities of virus circulating in blood of *Mavs*^*−/−*^ mice 14 to 28 days after infection (top) and in bile from the gallbladder of *Mavs*^*−/−*^ mice 7 days after infection (middle). Also included for reference are the previously published density of virus in feces of an infected DKO mouse (9) and previously published densities of virus in chimpanzee samples (red) (5) (bottom).

To ascertain whether mice express host factors essential for eHAV biogenesis, we studied AML12 cells (25). These are derived from hepatocytes of mice transgenic for human transforming growth factor alpha (TGF-α) and support replication of human HAV. Virus present in supernatant fluids of infected AML12 cultures was predominantly quasi-enveloped, with 88.6% of the virus banding at a density of ~1.087 g/cm^3^ in an iodixanol gradient (Fig. 5A). This closely matches the density of eHAV released from human hepatocyte cultures (Fig. 3D). Since TGF-α is unlikely to be involved in eHAV biogenesis, this indicates that mice express host factors required for eHAV biogenesis. Consistent with this, we found previously that virus recovered from extracts of infected mouse liver is predominantly membrane associated, possessing a buoyant density (~1.125 g/cm^3^) only slightly greater than that of eHAV in chimpanzee serum (9).

We next sought to characterize the buoyant densities of virus in murine serum (originating from the basolateral membrane of hepatocytes). We studied virus in serum from infected *Mavs*^*−/−*^ mice, collected prior to the development of anti-HAV antibody. These knockouts do not develop hepatic inflammation or ALT elevations and thus mimic the asymptomatic, prodromal phase of hepatitis A in humans when eHAV circulates in blood (5). Surprisingly, these results suggested the presence of two distinct virus populations: a minor population banding at a density similar to that of eHAV in chimpanzee serum (fractions 11 to 13; 1.105 g/cm^3^) and a larger population banding at a density intermediate between that of eHAV and naked HAV particles (fractions 15 to 18; 1.167 g/cm^3^) (Fig. 5B, top). Virus in serum from infected *Ifnar1*^*−/−*^ *Ifngr1*^*−/−*^ (double knockout [DKO]) mice also banded predominantly at this intermediate density (see Fig. S1A in the supplemental material). Importantly, like eHAV, virions with intermediate density are still significantly less dense than HAV found in feces (Fig. 5B, bottom) and are thus likely to be associated with membranes. Surprisingly, however, treatment of virus recovered from the blood of infected DKO mice with 1% NP-40 did not alter its buoyant density in iodixanol gradients (see Fig. S1A). The nature of these virions is thus uncertain. We cannot exclude the possibility that they were derived from quasi-enveloped virions damaged by shearing forces during blood collection or centrifugation. However, explanations other than partial loss of the enveloping membrane need to be considered (see Discussion). The presence of a substantial virus “tail” in a gradient loaded with serum from an infected chimpanzee suggests that a small proportion (fractions 14 to 16) of virus circulating in chimpanzees may possess a similar intermediate density (Fig. 5B, top).

We similarly characterized the buoyant densities of virus present in samples of bile collected from the gallbladders of infected mice. This virus had been secreted across the apical hepatocellular membrane. Although virus in bile from infected *Mavs*^*−/−*^ mice banded over a range of densities, the greatest HAV RNA content was found in fractions with the density of naked, nonenveloped virions (fractions 18 and 19; 1.287 g/cm^3^) (Fig. 5B, middle). Virus in bile from infected DKO mice was similar, with the largest peak at 1.275 g/cm^3^ (see Fig. S1B). These density profiles resemble the patterns observed with bile acid treatment of gradient-purified eHAV (Fig. 4A) and are consistent with conversion of quasi-enveloped eHAV particles secreted into the bile to nonenveloped virions following exposure to the detergent action of bile acids in the canalicular lumen (Fig. 1B).

## DISCUSSION

Most evidence suggests that the major site of HAV replication is the liver, with virus shed in feces being produced in infected hepatocytes and released into the duodenum via the biliary system (7, 26–28). Our recent studies of HAV infection in mice with defective innate immunity provide additional support for this view (9). Viral RNA was detected only within the liver, where it was localized in hepatocytes, and not in the ileum or colon. We show here that virus circulating in the blood of these mice bands in iodixanol gradients at a significantly lower buoyant density than the naked, nonenveloped virus shed in feces, indicating that it is likely to be membrane associated (Fig. 5B, top; see also Fig. S1A in the supplemental material). Such virus is released into the circulation across the basolateral membrane of the murine hepatocyte. However, it was surprising to find two apparently distinct populations of virions in serum from infected *Mavs*^*−/−*^ mice: one with the density of eHAV in human and chimpanzee blood (~1.10 g/cm^3^) and a second, larger population with a density (~1.16 g/cm^3^) intermediate between those of eHAV and naked virions (>1.22 g/cm^3^). As mentioned above, this could have resulted from physical damage sustained by eHAV particles, during either the collection of blood or ultracentrifugation. Strikingly, treatment of intermediate-density virus from DKO mice with 1% NP-40 did not alter its density in iodixanol gradients (see Fig. S1A). These results are reminiscent of our previously published finding that NP-40 treatment of cell culture-derived eHAV does not fully shift its density to that of naked virions but rather shifts it to a similar intermediate density (1.15 to 1.17 g/cm^3^) (5). These results may be explained by the association of virus with cholesterol- and sphingolipid-rich, detergent-insoluble membrane microdomains (“lipid rafts”) (29). Importantly, exosomes appear to be enriched in such detergent-resistant membranes (DRM) (30, 31) and are likely to possess a mechanism of biogenesis similar to that of eHAV (6). An alternative possibility is that eHAV circulating in mice could bind serum proteins in a species-specific fashion. Additional studies, including detailed proteomic and lipidomic analyses, will be needed to fully characterize these intermediate-density virions.

In contrast to the more buoyant, membrane-associated virus present in serum, virus shed in feces by infected mice bands in iodixanol gradients at the buoyant density of naked picornaviral virions, thus recapitulating the situation in infected primates (Fig. 5B) (5, 9). A clear explanation for this difference in the buoyant density and membrane association of HAV particles in blood and feces has been lacking. The studies we describe here address three hypothetical explanations for this difference (Fig. 1B to D).

One possibility is that quasi-enveloped eHAV and naked, nonenveloped HAV virions egress from polarized hepatocytes via distinct export pathways: eHAV across basolateral membranes into the space of Disse (and thence into blood) and HAV across the apical membrane into bile (Fig. 1C). We have never favored such an explanation, as there is no definitive evidence for noncytolytic egress of nonenveloped HAV virions. Such virions always comprise a minor fraction of the virus found in supernatant fluids of infected cells and in some experiments are present in very small quantities (Fig. 2D). The exosome-like egress of eHAV from cells provides a mechanism for the proteinaceous viral capsid to traverse an intact plasma membrane. Poliovirus and other distantly related picornaviruses may exit cells in membranous blebs through a process of secretory autophagy (32). In contrast, there is no clear mechanism for egress of nonenveloped virus other than cell lysis. Thus, it seems likely that the relatively small numbers of nonenveloped virions found in cell culture supernatant fluids are derived from quasi-enveloped virions that have lost their membranes or from otherwise unapparent cell lysis.

To formally test the hypothesis that quasi-enveloped and nonenveloped forms of HAV undergo differential vectorial release from infected cells, we studied two previously described models of HAV infection in polarized cells. We confirmed previous studies showing that HAV is released almost exclusively from the apical membrane of polarized Caco-2 cells that are of gastrointestinal epithelial origin (Fig. 2C) (12). Almost all virus present in both apical and basolateral compartments of infected Transwell cultures of Caco-2 cells was quasi-enveloped (Fig. 2D). In contrast, after an initial delay of 24 h, we found that virus was released in a relatively balanced fashion from both apical and basolateral membranes of polarized HepG2-N6 cells that were of hepatocellular origin (Fig. 3C). These results differ somewhat from those of Snooks et al. (13), who reported that 95% of virions were released from the basolateral membrane of HepG2-N6 cells. This difference not withstanding, virus present in either compartment was again predominantly quasi-enveloped (Fig. 3D). Unlike Caco-2-derived virus, a small but distinct population of nonenveloped virions was present in both compartments of HepG2-N6 cells. The ratios of naked virions to quasi-enveloped virions were similar in the two compartments. It is interesting to speculate that the small fraction of nonenveloped virus (mostly absent in Caco-2 supernatants) might have been due to secretion of bile acids by these relatively well-differentiated hepatocytes (see below). Collectively, however, the data indicate that quasi-enveloped and nonenveloped HAV do not have vectorially distinct export pathways and thus argue against the hypothesis proposed in Fig. 1C.

Protein transport to the canalicular membrane is complex in hepatocytes, with glycosylphosphatidylinositol (GPI)-anchored and single-pass apical membrane proteins transported first to the basolateral membrane prior to undergoing transcytosis to the canalicular (apical) membrane (10, 33). It is likely that the unique nature of membrane protein trafficking in hepatocytes accounts for the differences observed in vectorial release of HAV in Caco-2 (derived from colonic epithelium) and HepG2-N6 (hepatocyte-derived) cell lines (12, 13). It is possible that use of the default hepatocyte export pathway might account for the reproducible delay that we observed in the apical release of eHAV from infected HepG2-N6 cells (Fig. 3C). Counihan and Anderson (14) have proposed recently that selective transcytosis of naked versus quasi-enveloped virions could result in apical release of naked virus by hepatocytes following initial secretion from the basolateral membrane (Fig. 1D). However, our data show an absence of selectivity and only a very limited capacity to mediate the transcytosis of either naked or quasi-enveloped HAV from basolateral to apical membranes of polarized HepG2-N6 cells (Fig. 3E). IgA antibodies specific for HAV have been shown to enhance both hepatocellular uptake and transcytosis of HAV (14, 34), but these antibodies develop late in the course of hepatitis A and are associated with a decrease, not an increase, in fecal shedding of virus (4, 35, 36). We conclude that hepatocellular transcytosis is unlikely to contribute materially to the fecal shedding of HAV.

This leaves the third hypothesis for consideration: that eHAV, secreted across the apical hepatocyte membrane into the biliary canaliculus, is stripped of its membranes by the detergent actions of bile acids (Fig. 1B). Early studies by Schulman et al. (8) found that virus in the bile (gallbladder aspirate) of experimentally infected chimpanzees had both the morphology and the density of nonenveloped virions (1.29 to 1.34 g/cm^3^ in CsCl). However, immune electron microscopy (IEM) was used to detect virus in those studies, and that would not have detected quasi-enveloped virions (5). Thus, while nonenveloped virions are well documented in bile, the possibility of the simultaneous presence of quasi-enveloped eHAV has not been excluded. Although our previous studies showed that eHAV is stable in porcine bile (5), it is important that this experiment did not reproduce the conditions to which HAV would be exposed in the proximal biliary canaliculus. Following secretion, bile acids are rapidly incorporated into mixed micelles, with phospholipids released from the luminal side of the canalicular membrane (11, 37). These phospholipids neutralize the detergent action of the bile acids, which is further reduced by the dilutional effects of water secretion during passage through the biliary system. Thus, detergent action is greatest at the luminal surface of the canalicular membrane across which both virus and bile acids are transported. Local concentrations of bile acids have never been measured at the luminal surface of the canalicular membrane (which must be traversed by HAV to gain access to the bile), but are likely to be very high.

The integrity of the eHAV membrane is destroyed by 1% NP-40. Capsid antigen can be detected by ELISA after such treatment, despite the fact that the density of the virus increases to only 1.15 to 1.17 g/cm^3^ as mentioned above (5). This matches the density of eHAV particles after suspension in a 93 mM concentration of TCA (Fig. 4A), a principal component of human bile (38). Thus, bile acids present in human bile are as effective as 1% NP-40 in destroying the integrity of the envelope. In contrast, 930 mM TCA promotes complete conversion of eHAV to nonenveloped virions (1.227 to 1.283 g/cm^3^). While high, the total concentration of bile acids within human gallbladder aspirates, well downstream of the canalicular membrane, reaches 170 to 180 mM (39). Thus, the transition to nonenveloped virion can be mediated entirely by the detergent action of bile acids and, as indicated above, is most likely to occur at or close to the canalicular membrane. Density gradient analysis of virus present in bile collected from infected DKO and *Mavs*^*−/−*^ mice confirmed this, as it revealed virus with a range of densities extending from that of eHAV to that of the naked particle (Fig. 5B, middle; see also Fig. S1B).

The phylogenetically unrelated hepatitis E virus (HEV) appears to be similarly wrapped in membranes when circulating in the blood during acute infection (40), and, like HAV, it is shed in feces as nonenveloped virions (41). The membrane of HEV may be similarly removed by bile acids within the proximal biliary tract. Both HAV and HEV are efficiently transmitted through the fecal-oral route. These viruses are likely to benefit from having two distinct forms of virions: a naked, nonenveloped species that is stable in the environment and optimized for transmission between hosts and a quasi-enveloped form that provides protection against neutralizing antibodies and thus facilitates spread within the host (6).

## MATERIALS AND METHODS

### Viruses.

A noncytopathic, cell culture-adapted variant of the HM175 strain of HAV, HM175/p16 (20, 21), was grown in Huh-7.5 cells (42), which were derived from a human hepatoma. Supernatant fluids were clarified by low-speed centrifugation and frozen in aliquots as virus stock.

### Cells.

Caco-2 cells, a human colon adenoacarcinoma cell line (HTB-37, obtained from the American Type Culture Collection), and Huh-7.5 cells (obtained from Charles Rice, Rockefeller University) were maintained in Dulbecco’s modified medium with Eagle’s salts (DMEM) supplemented with 10% fetal bovine serum (FBS), 2 mM glutamine, 100 U penicillin, and 100 µg/ml streptomycin at 37°C in a 5% CO_2_ atmosphere. A polarized subclone of HepG2 cells (clone N6) (13), a human hepatocellular carcinoma cell line, was kindly provided by David Anderson, Burnet Institute. The cells were grown in minimal essential medium (MEM) supplemented with 10% FBS, 2 mM glutamine, 15 mM HEPES, 100 U penicillin, and 100 µg/ml streptomycin in plastic flasks coated with collagen type I (Sigma Aldrich) at 37°C in a 5% CO_2_ atmosphere. FRhK-4 (fetal rhesus kidney) cells were grown as described previously (5). AML12 (ATCC CRL-2254) cells were grown in DMEM/F-12 medium (Life Science Technologies) supplemented with 10% FBS, 1× insulin-transferrin-seleniums (Gibco), 40 ng/ml dexamethasone, and 2 mM glutamine at 37°C in a 5% CO_2_ atmosphere.

To obtain cell culture monolayers on permeable membranes, cells were seeded on Transwell-COL tissue culture inserts (Corning) with a 4.7-cm^2^ growth area and 3.0-μm pore size. Caco-2 cells were seeded at a density of 3.2 × 10^5^ cells/cm^2^ and then cultured for at least 10 days prior to infection. HepG2-N6 cells were seeded at a density of 5 × 10^4^ cells/cm^2^ in the growth medium described above with the addition of 1% DMSO to promote polarity (13) and were incubated for at least 18 days prior to infection. The medium was replaced at intervals of 3 to 4 days. Confluent monolayers were used for virus infection. Monolayer cells on glass slides were used for antibody staining and were obtained by seeding 4-well chamber slides (Nunc Lab-Tec II CC2) at the same density as the Transwell cultures described above, followed by incubation for 10 days (Caco-2 cells) or 18 days (HepG2-N6 cells). HepG2-N6 cells were grown on glass slides in media containing 1% DMSO.

### Animal studies.

Mice lacking expression of both type I and type II interferon receptors (*Ifnar1*^*−/−*^ *Ifngr1*^*−/−*^ mice; referred to here as “DKO” mice) or the innate immune signaling adaptor protein mitochondrial antiviral-signaling protein (MAVS) (*Mavs^−/−^*) were infected by intravenous inoculation of either fourth- or fifth-mouse-passage HM175 HAV derived from infected *Mavs^−/−^* mouse liver (9). To characterize the density of virus circulating during acute infection, blood was collected from 2 to 4 mice by cardiac puncture using a 23-gauge needle at the peak of viremia and allowed to clot and serum was stored at −80°C. For density analysis, serum samples were pooled and subjected to isopycnic gradient centrifugation as described below. Bile was collected from the gall bladders of 2 to 3 mice using a 29-gauge needle at necropsy, pooled, and subjected to isopycnic gradient centrifugation. Procedures for monitoring infection in these mice were otherwise as described previously (9). All animal studies were carried out with the approval of the University of North Carolina Institutional Animal Care and Use Committee (IACUC).

### Assessment of cell polarity.

Caco-2 and HepG2-N6 cells grown on glass slides as described above were fixed in 4% paraformaldehyde (PFA) (Affymetrix) and then permeabilized in 0.25% Triton X-100 (Sigma Aldrich) for 15 min and blocked with 10% normal goat serum–phosphate-buffered saline (PBS) for 1 h at room temperature. Slides were incubated with polyclonal rabbit antibody to tight junction protein 1 (TJP1, or ZO-1) (Invitrogen) at a 1:100 dilution and mouse antibody to dipeptidyl-peptidase 4 (DPP4, or CD26) (Abcam, Inc.) at a dilution of 1:200 in 3% bovine serum albumin (BSA)–PBS overnight at 4°C (13, 43). Slides were washed three times in PBS and incubated with Alexa-labeled secondary Alexa-488 anti-rabbit antibody and Alexa-594 anti-mouse antibody (Invitrogen) in a solution of 3% BSA–PBS for 1 h at room temperature. Slides were then counterstained with DAPI (4′,6-diamidino-2-phenylindole), mounted with ProLong Gold Antifade Mountant (Life Technologies, Inc.), sealed, and examined with an Olympus FV1000 laser-scanning confocal microscope. Images of *xy* and *xz* sections were collected using a 30× objective. *z* sections were compiled from 0.41-μm-step images through each *x*-*y* section and assembled by the use of Volocity software (PerkinElmer).

### Assessment of monolayer integrity.

The integrity of Caco-2 and HepG2-N6 monolayers was assessed by measurements of permeability to fluorescein isothiocyanate-conjugated dextran (FITC-dextran; Invitrogen) (molecular mass, 500 kDa) (13, 43). Briefly, 1.5 ml of medium containing 100 μg/ml FITC-dextran was added to the apical chamber of Transwell-COL cultures in which the basolateral chamber contained 2.6 ml medium. Following 30, 60, or 90 min of incubation at 37°C, 100-μl aliquots of the basolateral fluid were removed and examined for fluorescence with a Synergy 2 (Bio-Tek) Multi-Mode microplate reader (excitation wavelength, 485 nm; emission wavelength, 528 nm). Diffusion into the basolateral chamber was calculated as the percentage of total FITC-dextran placed into the apical chamber per hour.

### Virus infections.

Monolayers grown on Transwell-COL inserts were inoculated with 1 × 10^7^ to 2 × 10^7^ genome equivalents (GE) of HAV (HM175/p16; predominantly eHAV) for 1 h at 36°C via the apical domain (Caco-2 cells) or the basolateral domain (HepG2-N6 cells). HepG2-N6 cells were washed twice with medium prior to inoculation to remove DMSO. Both cell types are permissive for HAV, but their modes of uptake of virus differ. Caco-2 entry is largely restricted to the apical plasma membrane (12), while viral entry occurs predominantly via the basolateral membrane of HepG2-N6 cells, consistent with uptake of virus from the bloodstream (13). Cells were extensively washed with medium on both the apical and basolateral sides and were refed with fresh medium. Day 0 supernatant samples were immediately collected, and on each successive day, the total apical and basolateral supernatants were collected for measurements of HAV RNA levels by quantitative real-time RT-PCR (RT-qPCR) and to determine the buoyant density of virions released into the medium. The integrity of HAV-infected Caco-2 and HepG2-N6 monolayers was measured at 7 and 4 days after infection, respectively, by the addition of FITC-dextran to the apical compartment, with samples collected from the basolateral compartment 60 min later for analysis, as described above. AML12 cells grown in a 6-well plate were inoculated with 1 × 10^7^ GE of HM175/p16 virus for 1 h at 36°C, extensively washed, and refed with fresh medium.

### Measurements of transcytosis.

HepG2-N6 monolayers grown on Transwell-COL inserts were washed twice with medium to remove DMSO and then inoculated with 1 × 10^8^ GE of gradient-purified eHAV or nonenveloped virions (HM175/p16) via the basolateral domain, followed by incubation at either 36°C or 4°C. Apical supernatants were collected at hourly intervals for measurement of HAV RNA by RT-qPCR, with the cells refed with prewarmed or precooled fresh medium. The integrity of the monolayers was measured 3 h after inoculation by the addition of FITC-dextran to the apical compartment, with samples collected from the basolateral compartment 60 min later for analysis as described above.

### RT-qPCR for HAV RNA.

RNA from culture supernatants and gradient fractions was extracted with a QiaAmp viral RNA isolation kit (Qiagen) (5). RT-qPCR analysis was carried out using an iScript One Step RT-PCR kit for probes and an iTaq Universal Probes one-step kit (Bio-Rad) with a CFX96 real-time PCR detection system (Bio-Rad). HAV RNA levels were determined by reference to a standard curve obtained with synthetic HAV RNA. Primers targeted sequences in the 5′ untranslated RNA segment of the genome (5′-GGTAGGCTACGGGTGAAAC-3′ and 5′-AACAACTCACCAATATCCGC-3′) (35). The 6-carboxyfluorescein/6-carboxytetramethylrhodamine (FAM/TAMRA) probe was 5′-CTTAGGCTAATACTTCTA TGAAGAGATGC-3′.

### Isopycnic gradient ultracentrifugation.

Cell culture supernatants were centrifuged at 2,000 rpm for 3 min to remove debris and then concentrated using a filtration device with a 100-K molecular weight limit (Amicon). The concentrated virus was loaded onto an 8% to 40% iodixanol (OptiPrep; Sigma Aldrich) step gradient in a 5-ml ultracentrifuge tube (Beckman Coulter, Inc.) and centrifuged at 37,000 rpm in a Beckman SW55i rotor for 24 h at 4°C in a Beckman Optina LE-80K ultracentrifuge (5). Approximately 20 fractions were collected from the top of the gradient, and the HAV RNA content of each was quantified by RT-PCR. The density of gradient fractions was determined by refractometry.

### Preparation of purified eHAV and HAV.

Culture supernatants from HAV-infected Huh-7.5 cells were centrifuged at 1,000 × *g* for 10 min at 4°C to remove debris and then further clarified by centrifugation twice at 10,000 × *g* for 30 min. Virus was concentrated by ultracentrifugation at 100,000 × *g* in a Superspin 640 rotor for 1 h at 4°C in a Sorvall Ultra-80 ultracentrifuge (5). The resulting pellet was resuspended in PBS and then loaded onto an 8% to 40% iodixanol step gradient and ultracentrifuged as described above. Approximately 20 fractions were collected, and the HAV RNA content and density of each fraction were determined. Fractions containing virus with the appropriate buoyant density (for eHAV, approximately 1.08 g/cm^3^, fractions 8 to 11; for nonenveloped HAV virions, 1.22 g/cm^3^, fractions 17 to 20) (5) were combined and subjected to filtration (Amicon) (100-K molecular weight limit) to remove iodixanol and to exchange the buffer to PBS. eHAV and HAV preparations were stored at −80°C until use.

### Treatment of eHAV with bile acids.

Stock solutions were prepared by dissolving chenodeoxycholic acid (CDCA; Sigma-Aldrich) in DMSO at 240 mM (100 mg/ml) and taurocholic acid (TCA, Sigma-Aldrich) in PBS at 186 mM (100 mg/ml) and stored at −20°C. Gradient-purified eHAV (approximately 5 × 10^7^ GE) was incubated in 24 mM CDCA–10% DMSO–PBS, 93 mM or 930 mM (freshly prepared) TCA–PBS, 10% DMSO–PBS, or PBS alone for 2 h at 37°C and then subjected to repeat isopycnic ultracentrifugation in iodixanol gradients as described above. The resulting fractions were assayed by HAV-specific RT-qPCR, and density was determined by refractometry. To assess the effect of bile acids on infectivity, fractions were combined and concentrated and bile acids were removed by diafiltration (100-kDa exclusion) followed by infectivity assay and antigen detection ELISA.

### HAV capsid antigen ELISA.

Virus samples (approximately 2 × 10^7^ GE) were captured by human convalescent antibody (JC virus plasma) coated on a 96-well polystyrene plate (5). Anti-HAV capsid monoclonal antibody K24F2 (Commonwealth Serum Laboratories) was incubated in the wells of the plate at room temperature for 1 h, followed by incubation with horseradish peroxidase-conjugated goat anti-mouse antibody (Southern Biotech) at room temperature for 1 h. Following the addition of substrate (3,3′,5′,5′-tetramethylbezidine), the optical density at 450 nm (OD_450_) was determined using a Synergy 2 (BioTek) microplate reader.

### Infrared fluorescent immunofocus assay.

Infectious HAV was quantified by an infrared fluorescent immunofocus assay (IR-IFA) in FRhK-4 cells (44). Briefly, virus samples were inoculated onto FRhK-4 cell monolayers in 12-well plates and allowed to adsorb for 2 h at 36°C prior to the addition of medium containing 1% methyl cellulose and 1% FBS. Following 10 days of incubation at 37°C in a 5% CO_2_ atmosphere, the medium was removed and cells were fixed with 4% PFA. The cell sheet was permeabilized with 0.25% Triton X-100, blocked with 10% normal goat serum–PBS, and incubated with anti-HAV mouse monoclonal antibody K32F2 (Commonwealth Serum Laboratories) diluted 1:600 in 3% BSA–PBS overnight at 4°C. The cells were washed with PBS and incubated for 1 h at room temperature with IRDye 680LT anti-mouse antibody (Li-COR Bioscience) (1 µg/ml)–3% BSA–PBS. To visualize viral replication foci, plates were scanned with an Odyssey infrared imaging system (Li-COR Bioscience).

### Statistics.

Statistical significance was assessed with various tests as described in Results, with calculations carried out using the Prism 6 for Mac OS X software package. A *P* value of less than 0.05 was considered significant.

## SUPPLEMENTAL MATERIAL

Figure S1  Iodixanol gradient profiles of virus present in serum and bile of infected DKO mice. (A) Serum from a DKO mouse at 29 days postinfection with a second-mouse-passage inoculum of HAV prepared from feces. The level of serum ALT was 192 U/liter, and anti-HAV was present. Gradient profiles of virus are shown before (top panel) and after (bottom panel) treatment of serum with 1% NP-40. (B) Virus in gallbladder bile from a DKO mouse collected 18 days after infection with fourth-mouse-passage virus. The level of serum ALT was 150 U/liter. Download Figure S1, TIF file, 0.3 MB
