# Supplementary figures and images for: Biliary Secretion of Quasi-Enveloped Human Hepatitis A Virus
Source: mBio. 2016 Dec 6;7(6):e01998-16. doi: 10.1128/mBio.01998-16 (PMC5142623; doi:10.1128/mBio.01998-16)

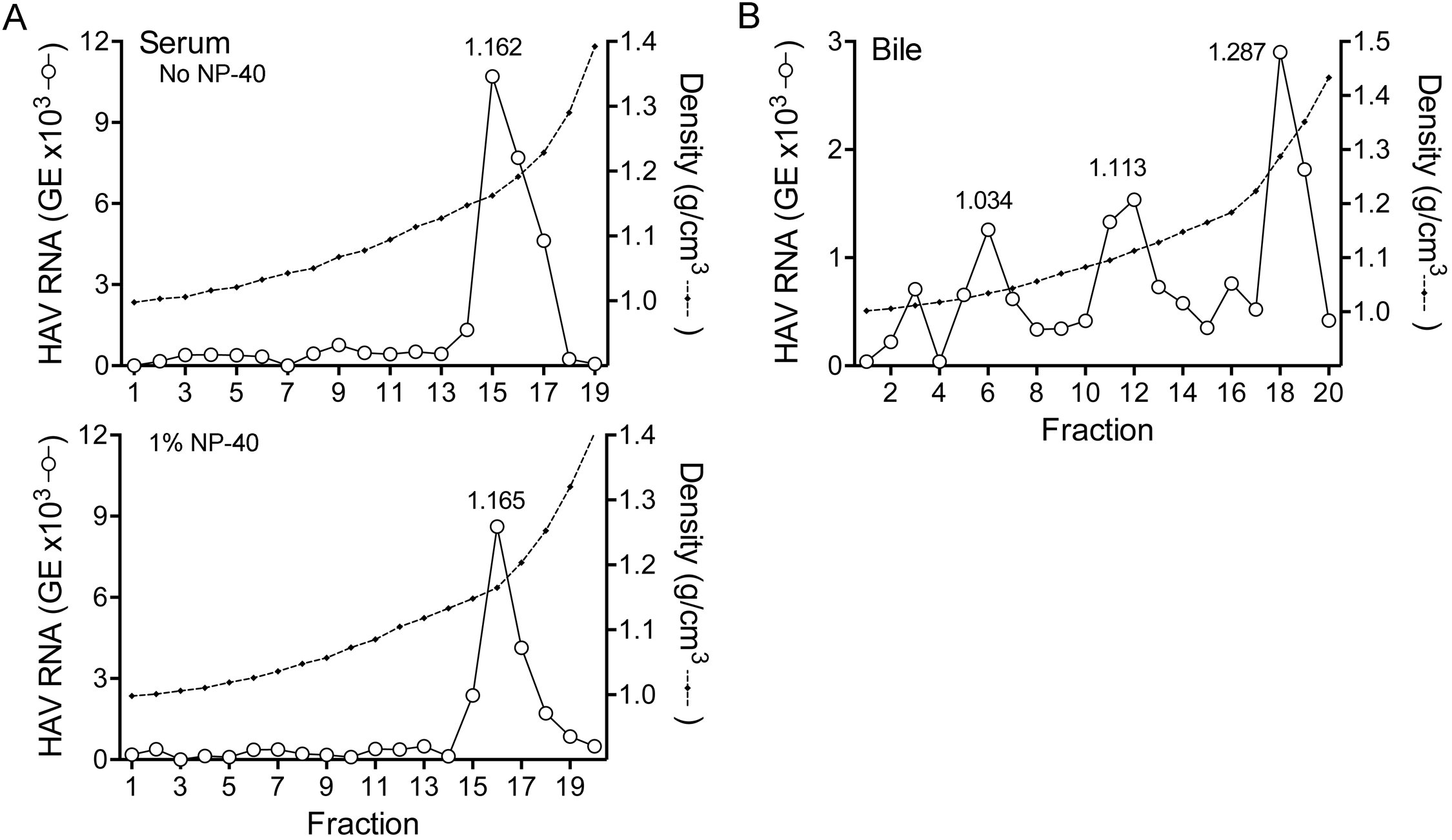

Supplement: Figure S1 — Iodixanol gradient profiles of virus present in serum and bile of infected DKO mice. (A) Serum from a DKO mouse at 29 days postinfection with a second-mouse-passage inoculum of HAV prepared from feces. The level of serum ALT was 192 U/liter, and anti-HAV was present. Gradient profiles of virus are shown before (top panel) and after (bottom panel) treatment of serum with 1% NP-40. (B) Virus in gallbladder bile from a DKO mouse collected 18 days after infection with fourth-mouse-passage virus. The level of serum ALT was 150 U/liter. Download [file mbo006163089sf1.tif]
